# Idiopathic focal organizing pneumonia mimicking malignancy

**DOI:** 10.11604/pamj.2020.36.256.23129

**Published:** 2020-08-07

**Authors:** Emrah Dogan, Utku Tapan, Ozge Oral Tapan, Turhan Togan, Özgür Ilhan Çelik

**Affiliations:** 1 Mugla Sitki Kocman University, Faculty of Medicine, Department of Radiology, Mugla, Turkey,; 2 Mugla Sitki Kocman University, Faculty of Medicine, Pulmonology, Mugla, Turkey,; 3 Mugla Sitki Kocman University, Infectious Diseases, Mugla, Turkey,; 4 Mugla Sitki Kocman University, Pathology, Mugla, Turkey

**Keywords:** Idiopathic focal organizing pneumonia, mimicking malignity, cryptogenic organizing pneumonia

## Abstract

Idiopathic FOP is a rare type of COP. What we know on this subject is made up of a few clinical cases published in recent years. Our patient was admitted to the hospital with an intermittent coughing complaint that worsens over time. Due to a suspicion of malignancy, a radiological evaluation was requested including a PET-CT and a transbronchial biopsy was performed. Until the last part of our algorithm, the patient profile was clinically and radiologically in favor of the diagnosis of malignancy but, in the end, the diagnosis of FOP was fixed with a follow-up decision. In conclusion, FOP is a relatively new entity that should be kept in mind in the differential diagnosis of malignancy.

## Introduction

Idiopathic focal organizing pneumonia (IFOP) is a rare presentation of cryptogenic organizing pneumonia (COP). Studies on this subject are relatively limited. FOP can often present imaging patterns similar to malignant lesions such as bronchogenic carcinoma, including positive results in contrast-assisted computed tomography and in positron emission tomography (PET-CT). Surgical resection is generally performed due to its asymptomatic clinical nature and its radiological similarity. More studies are needed to clarify the rate of idiopathic FOP and to clarify the uncertain causes of FOP, in particular the relationship between disease and infections [1,2]. We present a case of a 72-year-old patient with an idiopathic FOP and its radiological results.

## Patient and observation

**Figure 1:**
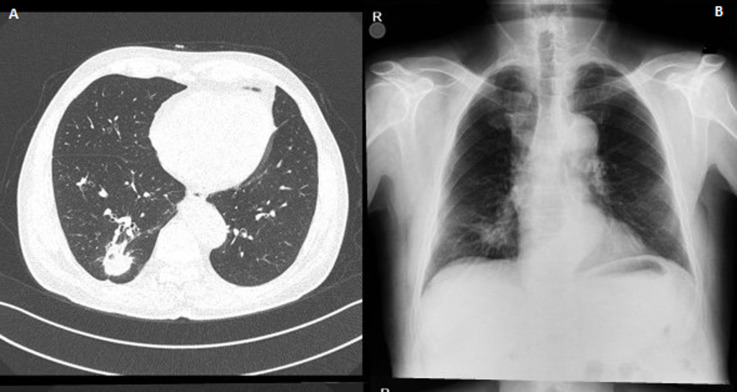
an irregular density was observed in the lower segment of the right lung on the chest X-ray (A), solid mass with speculated irregular contours mimicking malignancy, in the posterior basal segment of the lower lobe of the right lung (B)

**Figure 2:**
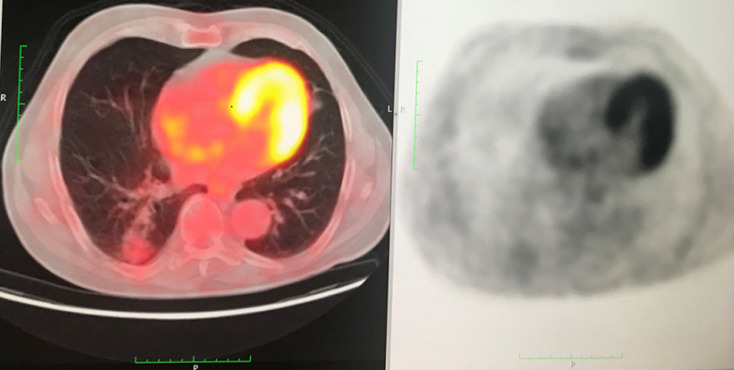
low grade FDG uptake in PET-CT (SUV: 1, 8)

**Figure 3:**
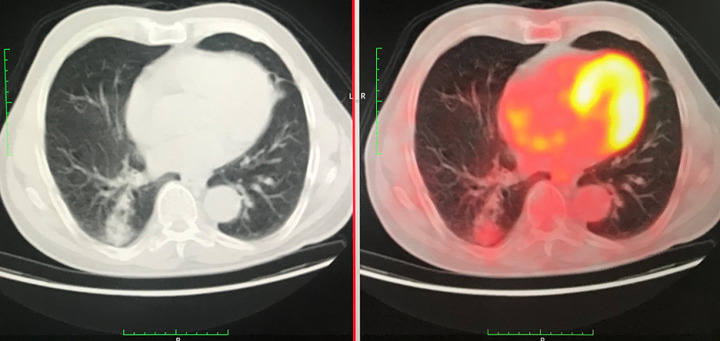
comparison of images taken by PET-CT and CT

A 72-year-old former “heavy” smoker (365 packs / year until 10 years ago) was referred to our service, presenting with a dry cough occurring at night and which has lasted for a year. The patient did not report any concept of shortness of breath, night sweats, fever or weight loss. No additional illnesses were found in the medical history. During the clinical pulmonary examination, lung sounds were normally perceived on both sides. Lung function tests were also normal. Laboratory tests involving white blood cell count (5.31x10^3^/ L), sedimentation rate and CRP were normal. Radiologically; an irregular density was observed in the lower segment of the right lung on the chest X-ray. A thorax CT was requested. It showed a solid mass of 27x36 mm with speculated irregular contours, in the posterior basal segment of the lower lobe of the right lung. Irregularity in the contour of the inner wall of the adjacent bronchi and bronchiectasis in the neighboring areas have also been observed. Furthermore, there was a distortion of the adjacent pleura. Ancillary radiological results supported the theory of the chronic process and therefore malignancy was considered (Figure 1). Due to the suspicion of a malignant process, a bronchoscopy was performed and a PET CT was requested. In PET CT; Low grade FDG (Fluoro-2-deoxy-2-d-glucose) uptake was present. [SUV (standardized uptake value): 1.8] (Figure 2, Figure 3).

**Figure 4:**
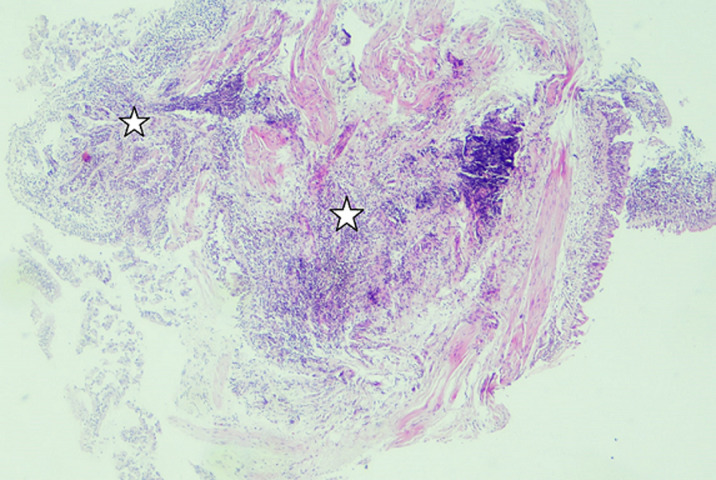
moderate inflammation appearance in the wall of the bronchi under the light microscope

**Figure 5:**
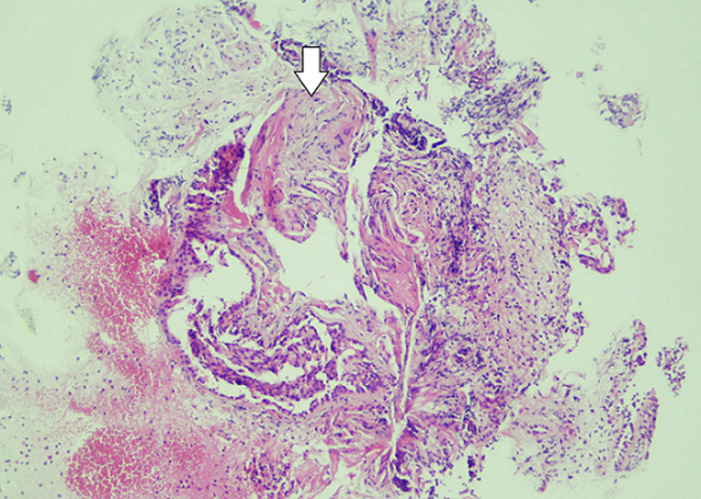
patchy involvement with loose fibroblastic plugs in the second biopsy

**Figure 6:**
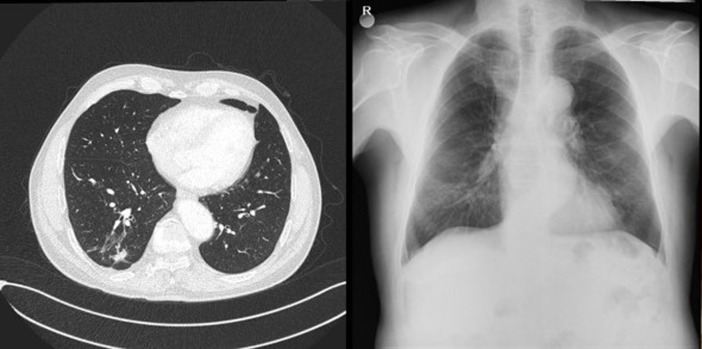
significant regression in control X-ray graphy and CT images

In bronchoscopy, the left bronchial system was open at the subsegmental level. The mucosa of the right lower lobe bronchi was coarse and irregular. Lower lobe posterior basal segment bronchus (RB 10) was successfully reached. Brushing was done from the apical region of this area. Broncho aspiration samples were obtained and sent for ARB (acid-resistant bacillus) research. No endobronchial lesion was observed. Transbronchial biopsy was performed. In the histopathological examination of the small bronchial biopsies under the light microscope; moderate inflammation composed of predominantly lymphocytes, plasmocytes and a few neutrophils and eosinophils were seen in the wall of one bronchi. Reactive changes were seen in the bronchial epithelium (Figure 4). In the second biopsy; there was a patchy involvement composed of a loose fibroblastic plug filling an air space. This plug had typically elongated, spindle and stellate fibroblasts embedded in a pale-staining matrix. Some fibrin deposits were seen around (Figure 5). Final interpretation of pathologist was; with all of these histopathological findings it was thought that the case may be compatible with Bronchiolitis Obliterans Organizing Pneumonia (Or if the case is idiopathic; Cryptogenic organizing pneumonia) if clinical and radiological findings support it. There was significant regression in the lesion on 2-month follow-up radiographs without any treatment (Figure 6). No predisposing factor to explain the patient's clinic was detected. Organizing pneumonia diagnosis was evaluated as cryptogenic. Pathology, clinical and radiological findings were in favor of the COP.

## Discussion

COP is a disease of unknown etiology characterized by granulation tissue blocking the alveoli. In a joint report of the American Thoracic Society (ATS) and the European Respiratory Society (ERS) which last revised in 2013, COP was included in the class of idiopathic interstitial pneumonia [3]. Organizing pneumonia is defined as secondary organizing pneumonia (SOP) when it is due to an etiology. Organizing pneumonias sometimes manifest as solitary lesions known as focal organizing pneumonia (FOP). Therefore, currently the studies separate FOP into Focal COP (FCOP) and focal secondary organizing pneumonia (FSOP) [1,2]. Our case was terminologically included in the FCOP group. Clinically; FOP presents with symptoms similar to organizing pneumonia. Usually starts with flu-like symptoms. The most common symptoms are fever, fatigue, dry cough, shortness of breath, loss of appetite and weight loss. Hemoptysis, profuse sputum, chest pain, joint pain and night sweats have been reported but are less common [4,5]. FOP patients are more asymptomatic than OP patients.

In physical examination, rare rales may be heard in the affected areas [4]. Our patient presented only an isolated cough. Radiologically, the common appearance of OP is in the form of patch-like migratory opacities that extend to the pleura. It can also be observed in the form of ground-glass opacities, air bronchograms or consolidations. The least common patterns are interstitial opacities, small overlapping alveolar opacities, diffuse bilateral infiltration, and solitary focal lesions [5,6]. The reverse halo sign was described as a special radiological discovery for COP and was almost specific for this disease until 2019 with the onset of the corona virus pandemic. New data has shown that there are findings that can be confused with Covid-19 pneumonia [7,8]. Unlike the models described above, those of POF imitate malignancy with spiculated or irregular contours and a location close to the peripheral region [2]. Unfortunately; there is no specific radiological manifestation that can distinguish FOP from bronchogenic carcinoma [9].

Histopathologically, FOP does not differ from OP. It is diagnosed by granulation tissue buds consisting of fibroblasts, collagen, fibrin exudate and alveolar ducts (Masson bodies). Therefore, it is still controversial whether idiopathic FOP is specifically a clinical pathology [2,10]. The diagnosis of COP without biopsy and using only the clinic has increased in recent years. So; detailed interrogation, clues of the underlying disease, physical examination, bacteriological and immunological tests should be used for the differential diagnosis. The COP remains a diagnosis of exclusion [4,11]. In cases defined as FCOP, clinical diagnosis is difficult, unlike other models of COP. Interventionally, transbronchial lung biopsy or a video assisted biopsy may be helpful in making the diagnosis. The transbronchial biopsy performed during a bronchoscopy can diagnose 69% of cases [2,10]. Unfortunately, unresolved cases are generally operated on [1,2,4,10]. The treatment, similar to that of COP, responds remarkably to corticosteroids. Clinical symptoms improve within 48 hours. The complete resolution of pulmonary infiltrates on radiographies generally takes several weeks [1,2,4].

## Conclusion

Our patient is an atypical case since the clinical symptomatology is non-specific, the radiological characteristics imitate malignancy and the evolution is marked by a spontaneous regression. Similar cases have been reported in the literature, diagnosed without biopsy or PET-CT and regressing spontaneously. FOP is a rare manifestation of OP that can be misdiagnosed as a malignant tumor. Currently, it is defined only as a presentation of the OP with a different radiological form. The causal relationship with smoking, the idiopathic PO/secondary PO ratio and the clinical profile that can distinguish it from other diseases of the COP group, are interesting perspectives to study in the future. FOP is a candidate to be defined as a different entity with specific characteristics.
